# Mathematical Modelling of the Molecular Mechanisms of Interaction of Tenofovir with Emtricitabine against HIV

**DOI:** 10.3390/v13071354

**Published:** 2021-07-13

**Authors:** Sara Iannuzzi, Max von Kleist

**Affiliations:** 1 Project Group 5 “Systems Medicine of Infectious Disease”, Robert Koch Institute, Nordufer 20, 13353 Berlin, Germany; kleistm@rki.de; 2 International Max-Planck Research School “Biology and Computation” (IMPRS-BAC), Ihnestrasse 63-73, 14195 Berlin, Germany

**Keywords:** NRTI, NNRTI, DEC, dNTP, DDI, HIV, PrEP, intracellular, HAART, Loewe, TFV

## Abstract

The combination of the two nucleoside reverse transcriptase inhibitors (NRTI) tenofovir disoproxil fumarate (TDF) and emtricitabine (FTC) is used in most highly active antiretroviral therapies for treatment of HIV-1 infection, as well as in pre-exposure prophylaxis against HIV acquisition. Administered as prodrugs, these drugs are taken up by HIV-infected target cells, undergo intracellular phosphorylation and compete with natural deoxynucleoside triphosphates (dNTP) for incorporation into nascent viral DNA during reverse transcription. Once incorporated, they halt reverse transcription. In vitro studies have proposed that TDF and FTC act synergistically within an HIV-infected cell. However, it is unclear whether, and which, direct drug–drug interactions mediate the apparent synergy. The goal of this work was to refine a mechanistic model for the molecular mechanism of action (MMOA) of nucleoside analogues in order to analyse whether putative direct interactions may account for the in vitro observed synergistic effects. Our analysis suggests that depletion of dNTP pools can explain apparent synergy between TDF and FTC in HIV-infected cells at clinically relevant concentrations. Dead-end complex (DEC) formation does not seem to significantly contribute to the synergistic effect. However, in the presence of non-nucleoside reverse transcriptase inhibitors (NNRTIs), its role might be more relevant, as previously reported in experimental in vitro studies.

## 1. Introduction

Nucleoside analogs denote a broad class of inhibitors that are successfully used in the treatment of cancers and many viral infections, such as Hepatitis B and C, Herpes viruses and HIV [1,2]. Moreover, nucleoside analogs are investigated for treatment of Ebola virus [3,4], as well as SARS-CoV-2 [5] and other RNA viruses. Most nucleoside analogs inhibit viral polymerase, which is necessary to maintain and multiply viral genomic information. Nucleoside analogs that target the RNA-dependent DNA polymerase of HIV are called nucleoside reverse transcriptase inhibitors (NRTIs) [6]. Like all other nucleoside analogs, NRTIs are administered as prodrugs. After uptake into HIV infected target cells [7], they undergo intracellular phosphorylation to form an analogue of (deoxy) nucleoside triphosphate [2]. The triphosphorylated NRTIs compete with the natural substrates for reverse transcriptase (RT) mediated incorporation into the nascent viral DNA. Once incorporated, the drugs block further polymerase activity because they lack the necessary chemical group that allows the further attachment of the next incoming nucleotide [8]. However, after incorporation, the drug can also be excised from the terminated primer after some time [9]. Therefore, the molecular mode of action of NRTIs can be viewed as a transient slowing down, rather than irreversible termination, of viral DNA polymerization [10].

NRTIs are typically given in combination. For example, highly active antiretroviral treatments (HAART) for HIV-1 infection typically consist of a two-drug NRTI combination plus a drug from a different class (e.g., protease-, integrase- or non-nucleoside reverse transcriptase inhibitors). Moreover, since 2013, the two-drug NRTI combination Truvada (Tenofovir disoproxil fumarate; TDF and Emtricitabine; FTC) is available for pre-exposure prophylaxis (PrEP) to prevent HIV infection [11]. Since 2019, Truvada is patent-expired, causing a massive drop in costs, which now make its broad use as PrEP cost-efficient in most regions of the globe [12,13]

Tenofovir disoproxil fumarate (TDF) is a prodrug of tenofovir (TFV), which is an adenosine monophosphate analogue. TDF is transformed into TFV after first-pass through the liver. TFV is the (monophosphorylated) circulating form of the drug, which, when taken up by cells, is twice phosphorylated to form TFV-DP, the active moiety [14]. The intracellular TFV-DP is an analogue of deoxyadenosine triphosphate (dATP). It thus competes with cellular dATP for positions of ‘A’ in the genetic code of the virus. Emtricitabine (FTC), on the other hand, is a deoxycytidine analogue. After cellular uptake and tri-phosphorylation, emtricitabine triphosphate (FTC-TP) is formed, which competes with cellular deoxycytidine triphosphate (dCTP) for incorporation at positions of ‘C’ in the genetic code of the virus [14]. Both drugs therefore inhibit reverse transcription at distinct positions (‘A’ vs. ‘C’ in the genome).

The two-drug combination Truvada (TDF + TFC) has been shown to be highly efficacious. Moreover, based on in vitro studies, it has been proposed that TDF and FTC act synergistically [15,16,17].

However, the mechanisms involved in the synergistic effect of the drug combination are still a matter of debate. Different models for assessing drug interactions exist [18,19] and remain an active field of research [20,21]. Typically, methods for analyzing drug–drug interaction are descriptive. They aim at explaining an efficacy surrogate in terms of an interaction metric. However, it is unclear whether the putative interaction of drugs is related to a direct interaction (e.g., drug A alters the concentration or potency of drug B), or a mere interaction of the targeted processes [22,23,24]. Moreover, the interpretation of combination screens is dependent on the metric used [25,26].

For Truvada, two mechanisms of direct interaction have been proposed: It has been proposed that FTC-TP alters the removal of TFV-TP from a terminated primer through the formation of a dead-end complex (DEC) [16,17]. Moreover, both drugs may alter the concentrations of their endogenous competitors (dCTP and dATP respectively) [27,28], which would increase their respective potency.

However, a direct link between the hypotheses of direct interaction and the observed interaction metrics has not been made.

In this work, we want to investigate if, and under which conditions, the hypothesized direct interactions between FTC-TP and TFV-DP can explain the deduced interaction metrics. For this, we extend a mathematical model of the molecular mechanisms of action of NRTIs [10], which is parameterized with physiological data and has been validated with clinical data [29]. In a previous study, we demonstrated how this model can be used to predict the concomitant pharmacological action of NRTIs if they do not interact directly [30]. In this work, we extend the model for the two types of direct interaction (DEC formation and effects on dNTP pools) and assess for realistic parameter ranges if the proposed hypotheses can explain the observed levels of synergy between the drugs.

## 2. Materials and Methods

### 2.1. Molecular Mechanism of Action (MMOA) Model

A previously defined and validated model [29] for the molecular mechanism of action (MMOA) was used to compute the inhibition of reverse transcription (ε) by NRTIs, and from there the inhibition of cell infection (η).

The model is motivated by two observations: (i) DNA polymerization is the rate limiting step during reverse transcription [31], and (ii) the amount of RT enzymes greatly exceeds the number of RNA templates (approximately 250-to-2; [32]).

**Figure 1:**
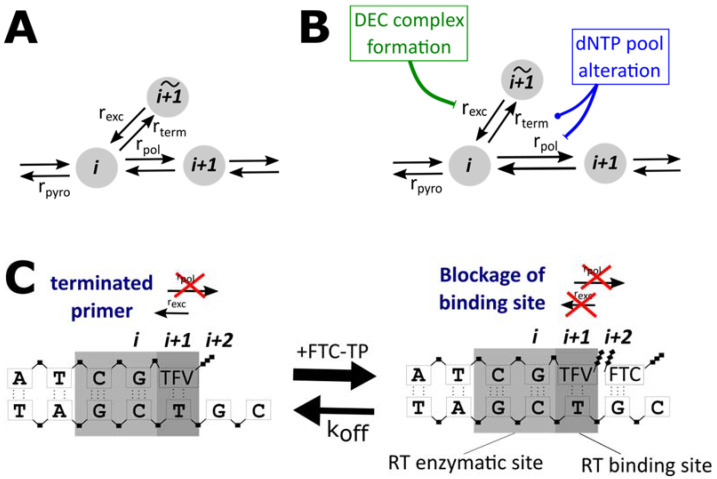
Model of the molecular mechanisms of action of NRTIs and direct drug interactions; (**A**) Basic model. A primer sequence of length i may either be prolonged by the polymerase reaction $r_{pol}\;(i+1$) or may be terminated by the incorporation of an NRTI-TP $r_{term}\;\left(i+1\right)$, depending on the nucleotide to be incorporated at position (i+1). An incorporated NRTI may be excised with rate $r_{exc}\;\left(i+1\right)$. Moreover, the primer of length (i+1) may be shortened by the pyrophosphorolysis reaction $r_{pyro}\left(i+1\right)$. (**B**) Direct drug interactions. We consider two types of direct drug interactions. Firstly, NRTIs may alter the pools of endogenous dNTPs, e.g., by binding to the respective intracellular kinases and blocking them. This increases $r_{term}\;\left(i+1\right)\;$ and decreases $r_{pol}\;\left(i+1\right).$ Secondly, it was hypothesized that FTC-TP may induce a dead-end complex after incorporation of TFV-DP into the primer, which alters the excision of TFV-DP from the terminated primer. (**C**) Mechanisms of dead-end complex formation: FTC may bind to a TFV-DP terminated primer at position (i+2), opposite of a ‘G’ in the template sequence. This binding blocks access to the incorporated TFV-DP and thus prevents excision unless FTC-TP unbinds.

The model explicitly considers reverse transcriptase (RT)-mediated polymerization of nascent viral DNA. The activated NRTI-triphosphates (NRTI-TPs) interfere with polymerization by competing with endogenous deoxynucleotide triphosphates (dNTPs) for incorporation into viral DNA. Upon integration of the NRTI-TP into the extending primer, the lack of a hydroxyl group impairs incorporation of the next incoming nucleotide, resulting in a halt of the polymerization process. This state can be reversed by excision of the NRTI-TP from the primer, as depicted in Figure 1A.

All in all, the model allows us to compute the mean first hitting time, i.e., the time required to complete viral DNA polymerization in the absence $T_{0\to N}\left(\emptyset \right)$ or presence $T_{0\to N}\left(I\right)$ of NRTI-TPs, where N denotes the length of the viral DNA. All parameters of the model can be derived from pre-steady state kinetic assays as outlined in [29].

The quantity of interest in describing the pharmacological effect is the residual reverse transcription in the presence of NRTI-TP, which can be computed from the mean first hitting times $T_{0\to N}$:

(1) $$1-\varepsilon \left(I\right)=\frac{T_{0\to N}\left(\emptyset \right)}{\begin{array}{c} T_{0\to N}\left(I\right) \end{array}}.$$

In previous work [29], the polymerization process was described as a Markov Jump Process, which allowed one to compute the mean first hitting time analytically using the recursion

(2) $$\;\;T_{0\to N}=\overset{N-1}{\underset{i=0}{\sum }}\;T_{i\to i+1}$$

where *i* denotes the position along the primer and $T_{i\to i+1}$ denotes the *expected time* to extend this primer by one base. As depicted in Figure 1A, four main reactions are considered by the model: the shortening of the primer by pyrophosphorolysis $r_{pyro}$, the extension of the primer through the polymerase reaction $r_{pol}$, the blockage of the primer by incorporation of an NRTI-TP $r_{term}$ and the excision of the NRTI-TP from the blocked primer $r_{exc}$.

The polymerization rates $r_{term}$, $r_{pol}$ are defined following Michaelis–Menten kinetics with competitive inhibition.

(3) $$\;r_{term}\left(i+1\right)=\frac{k_{term}\cdot \left[I\right]}{K_{D,I}\left(1+\frac{\left[dNTP\right]}{\begin{array}{c} K_{D,dNTP} \end{array}}\right)+\left[I\right]}$$

(4) $$\;r_{pol}\left(i+1\right)=\frac{k_{pol}\cdot \left[dNTP\right]}{K_{D,dNTP}\left(1+\frac{\left[I\right]}{K_{\begin{array}{c} D,I \end{array}}}\right)+\left[dNTP\right]}$$

The catalytic rate constants $k_{term}$ and $k_{pol}$ denote the incorporation of the NRTI-TP vs. the dNTP, respectively, at position (i+1) in the primer. The respective dissociation constants are denoted by $K_{D,I}$ and $K_{D,\;dNTP}$. For example, if the (i+1) position in the primer was an ‘A’, then, $k_{pol}$, $K_{D,dNTP}$ and [dNTP] correspond to the parameters for incorporation, and the concentration of dATP, whereas $k_{term}$, $K_{D,I}$ and [I] correspond to the parameters for incorporation and the concentration of the dATP analogue (TFV-DP), as depicted in Table 1.

The pyrophosphorolysis rate $r_{pyro}$ was set to 0.000898 (s^−1^) and the rates of NRTI excision of incorporated NRTIs in resting T-cells (r_exc_) were set to the values in resting CD4+ T-cells, e.g., 0.0016 [1/s] for TFV-DP, and to 0.00053 [1/s] for FTC-TP [29].

In Equation (3), the expected time to extend the primer by a single base is computed by considering the waiting times (τ) and the jump probabilities (ρ), where $\tilde{i+1}$ denotes the NRTI-TP blocked state.

(5) $$T_{i\to i+1}=\left(\tau_{\tilde{i+1}}\cdot \rho_{i\to \tilde{i+1}}+\tau_{i}+\rho_{i\to i-1}T_{i-1\to i}\right)\frac{1}{\rho_{i\to i+1}},$$

which relate to the reaction propensities via

(6) $$\begin{array}{c} \tau_{i}=\frac{1}{r_{pol}\left(i+1\right)+r_{pyro}\left(i\right)+r_{term}\left(i+1\right)},\;\tau_{\tilde{i+1}}=\frac{1}{r_{exc}\left(i+1\right)},\\ \rho_{i\to i+1}=r_{pol}\left(i+1\right)\cdot \tau_{i},\;\rho_{i\to i-1}=r_{pyro}\left(i\right)\cdot \tau_{i},\;\rho_{i\to i\tilde{+1}}=r_{term}\left(i+1\right)\cdot \tau_{i}.\; \end{array}$$

The terms $r_{pol}\left(i+1\right)$, $r_{term}\left(i+1\right)$ denote the incorporation of a natural dNTP at position (i+1) (polymerization reaction) vs. the incorporation of a nucleoside analogue into the nascent viral DNA at position (i+1) (termination reaction). The parameter $r_{pyro}\left(i\right)$ denotes the pyrophosphorolysis reaction, namely the rate at which a nucleoside is removed (excised) from the end of the primer. The parameter $r_{exc}\left(i+1\right)$ denotes the excision reaction, namely the rate at which an incorporated NRTI-TP is removed from the end of the primer. It is important to keep in mind that in the presence of two drugs targeting the same base, Equations (5) and (6) need to be adapted.

At position i=0 of the primer, we will have $r_{pyro}\left(0\right)=0$. Therefore, Equation (5) simplifies to Equation (7), where the term $T_{0\to 1}$ constitutes the start of the recursion.

(7) $$T_{0\to 1}=\left(\tau_{\tilde{1}}\cdot \rho_{0\to \tilde{1}}+\tau_{0}\right)\frac{1}{\begin{array}{c} \rho_{0\to 1} \end{array}}.$$

Finally, residual reverse transcription as quantified by Equation (1) can be used to calculate residual cell infection, which is typically quantified in cellular assays, and which can also be used as a drug efficacy parameter in multiscale modelling approaches [30]. Essentially, while residual reverse transcription considers the elongation of the time that RT requires to make proviral DNA, residual cell infection considers that the cell may clear essential viral components during that time. We previously derived a simple scaling [30] that captures the relationship between the two measures

(8) $$1-\eta \left(I\right)=\frac{1}{\rho_{\begin{array}{c} \emptyset ,RT \end{array}}+\frac{1-\rho_{\emptyset ,RT}}{\begin{array}{c} 1-\varepsilon \left(I\right) \end{array}}}$$

where $\rho_{\emptyset ,RT}$ = 0.5 [33] denotes the probability to succeed in reverse transcription in the absence of the drug (=the probability that viral building blocks are NOT eliminated before RT is finished). The validity of this approach has been demonstrated in [29] using top-down modelling on a disparate data set. Equation (8) produces a concentration-effect function that matches the classical Emax equation (with hill coefficient one), which interestingly has been confirmed independently by Shen et al. using replication assays [34]. Previous work [29] also revealed that the potency of NRTIs depends on a number of cellular factors. Most importantly for this study, the efficacy of NRTIs may depend on (i) the level of endogenous competing dNTP, (ii) as well as the rate of excision of the NRTI from the terminated primer.

### 2.2. Multiple Drugs

The previously introduced model can be adapted to compute the effect of drug combinations, as shown in Equation (9), where $I_{1},I_{2}$ are the two NRTIs.

(9) $$1-\varepsilon \left(I_{1},I_{2}\right)=\frac{T_{0\to N}\left(\emptyset \right)}{\begin{array}{c} T_{0\to N}\left(I_{1},I_{2}\right) \end{array}}$$

Within this manuscript, we focus on drugs that are analogs of different nucleosides. For example, tenofovir-diphosphate (TFV-DP) is a deoxyadenosine triphosphate (dATP) analogue, whereas emtricitabine-triphosphate (FTC-TP) is a deoxycytosine triphosphate (dCTP) analogue. It follows that the only change occurring when computing the effect of drug combinations (Equation (9)) is at the level of the corresponding base in the primer where one of the two drug presents can be incorporated. The rate $\;r_{term}$ will change according to the definitions given in Equations (3) and (4), where Equation (3) becomes non-zero and Equation (4) changes accordingly. With this change of rates, the hitting time will be computed as given in Equations (5) and (7). The residual cell infection can be computed akin to Equation (8):

(10) $$1-\eta \left(I_{1},I_{2}\right)=\frac{1}{\rho_{\emptyset ,RT}+\frac{1-\rho_{\emptyset ,RT}}{\begin{array}{c} 1-\varepsilon \left(I_{1},I_{2}\right) \end{array}}}=\frac{1-\varepsilon \left(I_{1},I_{2}\right)}{1-\rho_{\emptyset ,RT}\cdot \varepsilon \left(I_{1},I_{2}\right)},$$

where $I_{1},I_{2}$ denote the two drugs.

Several studies have investigated the effects of the combination of FTC-TP and TFV-DP both in vivo and in vitro [15,16,17,35,36] and reported synergistic effects. However, it is unclear whether the derived interaction metrics are related to a direct interaction of the two drugs, or a mere interaction of the targeted processes [22,23,24].

In previous work [10,30], it was assumed that the microkinetic parameters, as well as the concentration of endogenous dNTPs, are not affected by the presence of the two drugs.

A first obvious analysis is therefore the investigation of the output of common drug interaction metrics based on a ‘no direct interaction’ model. Secondly, we extend the model to probe hypothesized ‘direct interactions’ (Figure 1B), i.e., whether alteration of dNTP pools, as well as the formation of a dead-end-complex (DEC), may be mechanistic explanations of the observed interactions between TFV-DP and FTC-TP [15,16,17].

### 2.3. MMOA Modifications to Account for Direct Drug–Drug Interactions

#### 2.3.1. dNTP Pool Alterations

Chen et al. analyzed alterations in intracellular dNTP pools [37] after therapy with once daily oral 200/300 mg FTC/TDF therapy. The work indicated a decrease in dNTP pools within ~3 days after initiation of the combination treatment. For the four nucleotides the reductions were (in percent of basal levels) dCTP: 20%, dATP: 14% and dGTP: 19% and dTTP: 37%. In order to identify the corresponding intracellular NRTI-TP levels, we implemented the pharmacokinetic models linking the oral dosing schemes with the intracellular concentrations [29] and derived concentrations of 12.34 (FTC-TP) and 0.16 μM (TFV-DP), respectively. We then fitted a continuous function for the reduction factor *F_dNTP_* = Θ/(Θ + [*I*_1_ + *I*_2_]), where *I*_1_ + *I*_2_ denotes the concentrations of TFV-DP and FTC-TP in peripheral blood mononuclear cells (PBMCs), deriving Θ_dATP_ = 2.045, Θ_dACP_ = 3.125, Θ_dGTP_ = 2.932 and Θ_dTTP_ = 7.340 μM. The resulting fits are depicted in Supplementary Figure S1. The dNTP pool alteration model was then implemented as follows:

(11) $$\left[dNTP\left(I_{1},I_{2}\right)\right]=\left[dNTP\left(\emptyset \right)\right]\;*\;\frac{\Theta_{dNTP}}{\begin{array}{c} \Theta_{dNTP}+\left(I_{1}+I_{2}\right) \end{array}}$$

where [dNTP(∅)] denotes the basal dNTP concentration depicted in Table 1. The decrease in dNTP pools could lead to an increase in NRTI-TP incorporation (compare Equations (3) and (4)), which increases the potency of NRTIs and increases the mean first hitting time $T_{0\to N}\left(I\right)$ (also compare Figure 1B).

#### 2.3.2. Inhibition of the Excision Rate (DEC Formation)

Another plausible mechanism of interaction is the inhibition of TFV-DP excision from the terminated primer. The proposed mechanism is explained due to enhanced dead-end-complex (DEC) formation in the presence of an FTC-TP [16]. DEC formation would decrease the rate of excision of the incorporated nucleotide/analogue, which further increases the mean first hitting time $T_{0\to N}\left(I\right)$, Equations (5) and (6).

DEC formation is typically thought of as a mechanism by which the RT enzyme moves one step ahead, such that the nucleotide binding site can accept the next incoming nucleotide—however, without being able to attach it to the nascent viral DNA [38,39,40,41]. As long as the binding site is occupied by FTC-TP, there is no access to the incorporated TFV-DP for the excision reaction (Figure 1C). Hence, DEC formation can be viewed as a further prolongation of primer blockage.

The term $\tau_{exc}\left(TFV,\emptyset \right)$ (Equation (12)) refers to the waiting time for removal of the incorporated TFV-DP from the primer. The term $\tau_{exc}\left(TFV,FTC\right)$ denotes the (increased) waiting time when FTC-TP binds to the TFV-DP terminated primer. The overall contribution to the excision rate is given by Equation (13).

(12) τexc(TFV,∅)=1rexc τexc(TFV,FTC)=τexc(TFV,∅)∗(1+τFTC˜∗pi+1−>(i+1):FTC˜)

(13) $$\tau_{exc}\left(TFV,FTC\right)=\frac{1}{r_{exc\;}}\left(1+\frac{1}{k_{off}\left(FTC\right)}*\;\frac{\left[FTC\right]}{K_{D}\left(FTC\right)*\left(1+\frac{\left[dCTP\right]}{\begin{array}{c} K_{D}\left(dCTP\right)\; \end{array}}\right)+\left[FTC\right]}\right)$$

where $p_{\tilde{i+1->\left(i+1\right):FTC}}$ and $\tau_{\tilde{FTC}}$ denote the probability that FTC-TP binds to the TFV-DP terminated primer and $\tau_{\tilde{FTC}}$ denotes the duration of this binding.

The probability of FTC-TP binding was assumed to follow Michaelis–Menten kinetics, analogous to Equation (3). Note that FTC-TP binding to the TFV-DP-terminated primer can thus only occur when the nucleotide at position (i+2) in the primer should be a ‘C’ (opposite of a ‘G’ in the template, Figure 1C).

The duration of binding can be determined straight forward from the off-rate. However, this parameter is unknown. For the purpose of the analysis herein, we will assess the strongest possible effect of the DEC formation. That is, we will use the smallest possible value of $k_{off}\left(FTC\right)$.

It is generally assumed that the catalytic step (the incorporation of the NRTI or its analogue) is the rate limiting enzymatic step. This also justifies the use of the Michaelis–Menten formula for describing the enzyme kinetics (compare Equations (3) and (4)). Hence for FTC-TP, we have $k_{term}<k_{on},\;k_{term}<k_{off}.\;$From the definition of the dissociation constant $K_{D}=\frac{k_{off}}{\begin{array}{c} k_{on} \end{array}}$, we can derive $k_{off}=K_{D}*k_{on}$. Now, using the fact that binding is faster than catalysis, we have $k_{off}>K_{D}*k_{term}$. This means that if we set $k_{off}=K_{D}*k_{term}$ in the simulations, we have chosen a value for $k_{off}$ that is probably smaller than its ‘real’ (unknown) value. Thus, in our simulations, we may overpredict the DEC effect (we keep this in mind for the analysis). In all simulations we will use $k_{off}=K_{D}*k_{term}\;$= 1.0697 [s^−1^].

#### 2.3.3. Model Set-Up

A randomly generated sequence of 1000 nucleotides in length with equal frequency for each nucleotide was used. Residual cell infection data for the combination drugs (Equation (10)) were used to generate results in the absence and presence of TFV-DP, FTC-DP and their combination. The analysis was carried out for the MMOA model without direct drug interaction, as well as with the modified versions that regard altered dNTP pools, as well as DEC formation, either in isolation or in combination. All parameters of the model are depicted in Table 1. A nomenclature table can be found in the Supplementary Materials (Table S1).

### 2.4. Analysis of Interaction through Common Interactions Metrics

To assess the combined effect of FTC and TDF, the Python library ‘synergy’ was used [42]. This package allows to compute (i) Bliss independence, (ii) the combination index (CI), as well as (iii) Loewe additivity. Results are displayed with heatmaps generated with the visualization package seaborn (DOI:10.21105/joss.03021).

## 3. Results

### 3.1. Single Drug Dose Response Curve Is an EMAX Equation

**Figure 2:**
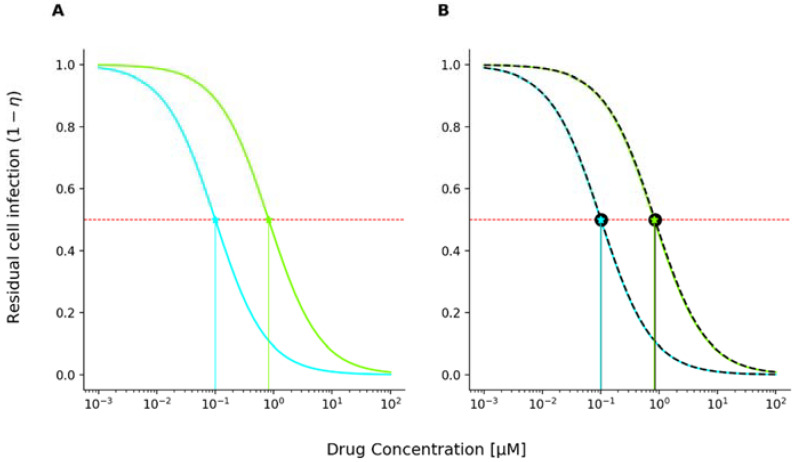
MMOA-predicted inhibition of cell infection; the red dashed horizontal line marks 50% inhibition. Projections (vertical lines) on the *x*-axis correspond to the fifty percent inhibitory concentration IC50. (**A**) Concentration-effect (inhibition of cell infection) curve for tenofovir diphosphate (TFV-DP, in cyan) and emtricitabine triphosphate (FTC-TP, in green) computed using the unmodified MMOA model. Data for the residual cell infection were obtained by solving Equation (8) for a heteropolymeric random sequence of length 1000 nucleotides with 25% respective dNTP content and parameters displayed in Table 1. (**B**) Concentration-effect curves for TFV-DP and FTC-TP with superimposed, fitted Emax curve (Equation (15)) with m = 1 and IC50 = 0.1 and IC50 = 0.84 μM for TFV-DP and FTC-TP, respectively (black dashed line).

Using the unmodified MMOA model, dose-effect curves for the single drug were generated by solving Equations (1) and (8) and are depicted in Figure 2A (green line: TFV-DP; cyan FTC-TP).

As can be seen, the concentration effect curve has a typical sigmoidal shape when plotted with a logarithmised *x*-axis (log concentration vs. effect). Subsequently, we fitted a classical Emax model (Equation (15)), which is typically used in pharmacodynamic analysis of concentration response relationships [34], to the data generated from the MMOA model:

(14) $$E=\frac{E_{max}{\left[C\right]}^{m}}{\begin{array}{c} IC_{50}{}^{m}+{\left[C\right]}^{m} \end{array}}$$

The resulting fits for tenofovir diphosphate and emtricitabine triphosphate are superimposed onto the MMOA concentration-response profiles in Figure 2B (black-dashed lines). In line with previous results [29], we obtain fifty percent inhibitory concentrations *IC*_50_ = 0.1 μM for TFV-DP and *IC*_50_ = 0.84 μM for FTC-TP, both with slope m=1. As can be seen in Figure 2B, the residual cell infection curves (dashed black line) are in agreement with results obtained by the Emax model. Note that Shen et al. [34] also independently found that the concentration-effect curve of NRTIs follows a classical Emax equation with slope one for the drug class of nucleoside reverse transcriptase inhibitors (NRTIs).

### 3.2. Modelling Combination Effects Using the MMOA Model

The MMOA model can be straightforwardly exploited to model the concomitant effects of two NRTIs on reverse transcription and cell infection using Equations (9) and (10).

#### 3.2.1. Unmodified Model

**Figure 3:**
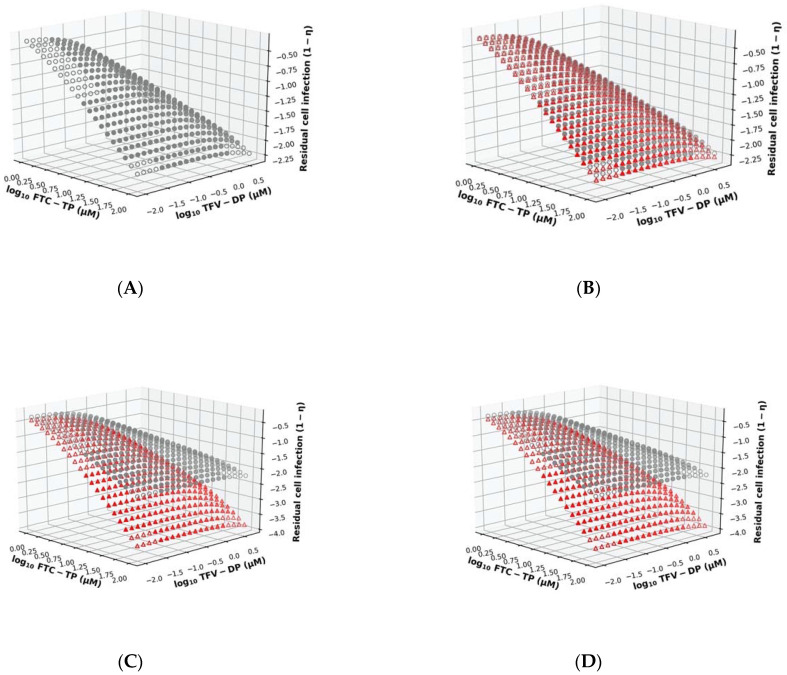
Drug combination surface plot. The *x*- and *y*-axis depict the concentration of TFC-TP and TFV-DP respectively. The solid dots mark clinically observed concentration ranges after once-daily oral administration of Truvada (300 mg TDF + 200 mg FTC). The *z*-axis depicts the residual cell infection computed from the MMOA model, Equation (10) (**A**). Residual cell infection computed from unmodified MMOA. (**B**) Residual cell infection computed from MMOA model with direct interaction of the drugs through dead-end-complex (DEC) formation (red dots), vs. unmodified model (grey dots) (**C**). Residual cell infection computed from MMOA model with direct interaction of the drugs through dNTP pool alteration (red dots), vs. unmodified model (grey dots) (**D**)**.** Residual cell infection computed from MMOA model with (DEC) formation and dNTP pool alteration (red dots) vs. unmodified MMOA (grey dots).

In the model, a single nucleotide analogue may become incorporated at its respective endogenous competitor site. For example, TFV-DP competes with dATP for incorporation into the nascent DNA at sites opposite of ‘T’ in the template sequence. Likewise, the two drugs TFV-DP and FTC-TP may independently compete with their respective endogenous competitors (dATP vs. dCTP) for incorporation opposite of ‘T’ (TFV-DP) or opposite of ‘G’ (FTC-TP) in the template sequence. Each of these incorporations may prolong the mean first hitting time $T_{0\to N}\left(I_{1},I_{2}\right)$, i.e., the time required for finishing reverse transcription, according to the summation in Equation (5). In Figure 3A, we analyzed the surface plot of the drug effect η (inhibition of cell infection) for the drug combination of TFV-DP with FTC-TP for concentrations ranging from 0.01–3.16 and 1–1000 μM, respectively, using the unmodified MMOA (20 × 20 grid). In the surface plot (Figure 3A), we also marked the concentrations of TFV-DP and FTC-TP that are typically achieved clinically in peripheral blood mononuclear cells (PBMC) after once daily oral administration of Truvada (200 mg FTC + 300 mg TDF) using solid dots. As expected, low concentrations of both drugs allow for residual cell infection. Within clinically achieved concentration ranges (filled dots), the effect of the drug combination FTC-TP+TDF-TP is largely reflected by the efficacy of FTC-TP. Next, we analyzed the effects of putative direct drug–drug interactions (Figure 1B) on the combination surface plot.

#### 3.2.2. DEC Formation

As a first analysis, we implemented the model for dead-end-complex (DEC) formation, in which FTC-TP may alter the excision of TFV-DP from the terminated primer (schematic in Figure 1C). Figure 3B shows the corresponding surface plot, where we show residual cell infection when TFV-DP and FTC-TP directly interact with regards to DEC formation (red triangles), in comparison to the unmodified MMOA (grey dots). As can be seen, DEC formation changes the surface plot only marginally.

#### 3.2.3. dNTP Pool Alterations

Next, we looked at the hypothesis that NRTIs alter the concentrations of endogenous dNTPs (details of the implementation are given in the Methods section). In Figure 3C, we can see that alterations of dNTP pools (red triangles) change the surface plot when compared to the unmodified MMOA model (grey dots). In particular, the reduction in cell infection occurs already at lower drug concentrations, leading to a higher potency of the drug combination. Moreover, the combination effect seems to be more pronounced in the FTV-TP direction, i.e., reduction of dNTP pools particularly boosts the potency of FTC-TP.

#### 3.2.4. dNTP Pool Alterations and DEC Formation

Finally, we modelled both direct interactions. The resulting surface plot is shown in Figure 3D. As can be seen, the surface plot for both direct interactions looks highly similar to the surface plot with the dNTP alteration only.

Next, we will investigate the output of interaction metrics for the combined drug effects.

### 3.3. Analysis Using the Combination Index

In the sequel, we apply a common metric that is commonly used to quantify drug–drug interaction to the MMOA predicted efficacy endpoints for the unmodified model, the model with dNTP alterations, the model with DEC formation and the model with dNTP alteration + DEC formation. In the discussion, we further motivate the choice of interaction metric.

**Figure 4:**
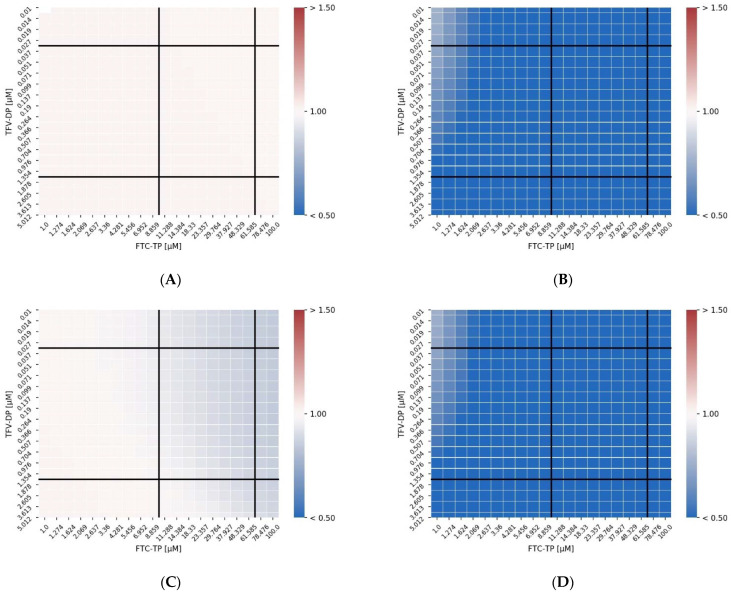
TFV-DP and FTC-TP interactions heatmaps (Combination Index). The concentrations that produced an effect classified as synergistic are shown in blue. The panels report results for the unmodified model and the other modifications applied. (**A**) control: unmodified MMOA model; (**B**) dNTP: reduced dNTP pools (**C**); exc: decrease in the excision rate; (**D**) dNTP+ exc: reduction of dNTP and decrease in the excision rate both incorporated in the MMOA model.

Figure 4A–D shows heatmaps of the combination index for the unmodified model, the model with dNTP interaction, the model with DEC complex formation and the combination of the two direct interactions. The combination index (CI) is a parametric method to assess whether a normalized concentration of drug *I*_1_ can be replaced by a normalized concentration of drug *I*_2_ (linear isobole),

(15) $$CI=\frac{I_{1}}{IC_{x,1}}+\frac{I_{2}}{IC_{x,2}}$$

where $I_{1}$ and $I_{2}$ are the concentrations of the two drugs, where $\eta \left(I_{1},I_{2}\right)=x$ percent inhibition is achieved and $IC_{x,1},\;IC_{x,2}\;$are the single drug concentrations, where *x* percent inhibition is achieved (e.g., the IC_50_ if *x* = 50%). *CI* < 1 indicates synergism, while *CI* > 1 indicates antagonism. In terms of interpretation, the combination index indicates whether the concentrations of a drug A should be increased/decreased in the presence of a drug B to obtain an equivalent overall effect. In other words, whether the potency of a drug is altered in the presence of a second drug.

In Figure 4, the solid black vertical and horizontal lines mark the clinically relevant ranges of FTC-TP and TFV-DP after once daily Truvada (300/200 mg oral TDF/FTC).

As can be seen in Figure 4A, there is no CI synergy detectable for the unmodified MMOA model across the analyzed concentration ranges. In contrast, we observe CI synergy between FTC-TP and TFV-DP when NRTIs alter the dNTP pools, Figure 4B. The level of synergistic interactions is much weaker for the DEC interaction model (Figure 4C) but occurs at high FTC-TP concentrations and within clinically relevant concentration ranges. The model with both interactions largely reflects the model where dNTP concentrations are altered, Figure 4D.

## 4. Discussion

Nucleoside analogs are a class of compounds that are used to treat a diverse array of viral infectious diseases, as well as cancers. When used against viruses, these drugs target viral polymerase, and in HIV, they are called nucleoside reverse transcriptase inhibitors (NRTI). The drugs tenofovir disoproxil fumarate (TDF) and emtricitabine (FTC) are the most frequently used NRTI backbone in highly active antiretroviral therapy (HAART) against HIV. Moreover, this two-drug combination is widely used as pre-exposure prophylaxis (PrEP) to protect high risk groups from HIV infection.

Clinical studies suggested that the two drugs do not affect each other’s pharmacokinetics [43]. On the other hand, top-down analysis of several in vitro studies suggested that TDF and FTC act synergistically [15,16,17]. It was furthermore suggested that the two drugs may interact intracellularly by affecting dNTP pools, or through dead-end complex (DEC) formation. However, the underlying mechanisms playing a major role in the occurrence of synergy remain to be defined. In this work, we analyse whether the suggested mechanisms of direct interaction between the drugs are able to explain the observed drug synergy. For analysis, we extended a previously published [10] model of the molecular mode of action (MMOA). Using this modified model, it is possible to predict the isolated drug effects, as well as their combined effect with or without direct drug-drug interactions. We then use the same metrics that were used in the original work [15,16,17] to analyze whether synergistic/antagonistic interactions would be expected at relevant concentration ranges.

Our analysis (Figure 3) indicates that dNTPs depletion may be the dominating mode of direct drug–drug interaction between TDF and FTC at clinically relevant concentration ranges. CI synergy can be observed at clinically relevant concentration ranges (Figure 4). DEC formation, on the other hand, had only marginal effects.

Interaction at the level of intracellular dNTP pools seems to be common among NRTIs and also a plausible mechanism of direct interaction. For example, Hawkins et al. [44] found that didanosine (ddI; a dATP analogue) significantly decreases dATP concentrations when co-administered with TDF. The study by Goicoechea et al. [45] analyzed the interaction of abacavir (ABC) with tenofovir (TFV). While they could not find evidence that one drug altered the concentrations of the other drug’s intracellular triphosphates, they did observe dNTP pool alterations for the drug combination [45]. In the study, it was observed that abacavir increases dATP concentrations, leading to an overall antagonism between the drugs. The proposed interaction took place at the level of intracellular phosphatases (dATP eliminating pathway).

The biochemical mechanisms underlying dNTP depletion in the drug combination TDF+FTC remain unknown. The activation (phosphorylation) mechanisms may be a likely source of interaction [46]. For example, if FTC interferes with the NTD kinase it may decrease dATP production, while as the same time, NTD kinase is not the rate-limiting step in the TFV to TFV-DP phosphorylation cascade [46]. In addition, Bousquet et al. [47] also showed that FTC and TDF increase the intracellular levels of their co-administered prodrugs, i.e., FTC increases intracellular TFV levels and TFV increases intracellular FTC levels through induction of transporters. It is not clear whether the elevation of the co-administered prodrugs also lead to an increase in the respective triphosphorylated moieties because nonlinear and rate limiting steps in the intracellular activation cascade are in place for both drugs [29,48]. However, elevating the level of prodrugs could also lead to an increase in the inhibition of the activation cascade of the natural substrates (e.g., the transformation of dA to dATP) as discussed above.

Interestingly, dNTP depletion was also discussed as a broad-spectrum mechanism of action of nucleoside analogues against coronaviruses [28].

With regards to DEC formation, enhanced formation of TFV-terminated template DNA complexes (DEC), in the presence of FTC-TP, have been observed [16]. However, no DEC was formed in FTC-terminated primers, in agreement with our modelling assumptions. It could also be observed in Feng et al. [16], from the FTC-TP concentration DEC formation curves, that the amount of DEC formed at relevant therapeutic FTC-TP concentration appears to be modest. However, the non-nucleoside reverse transcriptase inhibitor (NNRTI) efavirenz facilitated efficient formation of stable, DEC-like complexes by TFV-monophosphate (MP)-terminated DNA. A subsequent study by Kulkarni et al. [17] also reports that DEC formation is actually more likely with addition of an NNRTI than with FTC-TP. A possible underlying mechanism may be the fact that NNRTIs alter the conformational flexibility of the RT enzyme [49,50,51], thus potentially ‘locking’ it in the DEC state.

Two drug interaction metrics (and various version of it) are usually used to assess synergy vs. antagonism. These metrics either measure independence of drug target processes, such as Bliss independence, or they measure changes in drug potency. The latter denotes a variety of methods, which can be derived from one another, such as isobologram analysis, Loewe additivity and the combination index [18]. In essence, isobologram analysis draws a line for a specific level of efficacy, assuming that drug one can be replaced by a normalized concentration of drug two. The normalization refers to the ratio of drug potencies when each drug was given on its own. If the actual efficacy for the combination can be achieved below this line, synergy is observed (otherwise, antagonism), i.e., if the observed efficacy for a drug combination requires lower concentrations than expected, we have synergy.

While the isobologram analysis is visual, Loewe additivity formalizes this concept for arbitrary but known concentration-effect functions using the following formula:

(16) $$Loewe=\frac{I_{1}}{\eta_{1}^{-1}\left(\eta \left(I_{1},I_{2}\right)\right)}+\frac{I_{2}}{\eta_{2}^{-1}\left(\eta \left(I_{2},I_{1}\right)\right)}$$

where $\eta \left(I_{1},I_{2}\right)$ denotes the effect of the drug combination with concentrations $I_{1}$, $I_{2}$ and $\eta_{1}^{-1}$ denotes the inverse dose response function for only compound one; i.e., the concentration of compound 1, which would produce effect $\eta \left(I_{1},I_{2}\right)$ if it was given alone. Consequently, *Loewe* < 1 indicates synergy, while *Loewe* > 1 indicates antagonism.

The combination index is a special case of Loewe additivity where the concentration response profile is an Emax equation. Hence the denominator can be replaced with IC_x_. Because we observe an Emax equation (Figure 2), we focus on the combination index in our analysis.

Bliss independence, on the other hand, assesses a different endpoint; the metric tests whether the inhibited processes of the respective drugs are independent with regards to the efficacy endpoint. Bliss independence is defined as

(17) $$Bliss=\left(1-\eta \left(I_{1}\right)\right)\cdot \left(1-\eta \left(I_{2}\right)\right)-\left(1-\eta \left(I_{1,2}\right)\right),$$

i.e., Bliss > 0 indicates synergy, while Bliss < 0 indicates antagonism. In fact, for TFV-DP + FTC-TP we observe Bliss antagonism (Supplementary Figure S2). This observation is related to the fact that both drugs act on the same pathway (reverse transcription). Therefore, they are naturally not *Bliss independent*. The type of Bliss antagonism observed is similar to that observed for ‘serial targets’ [52,53].

## 5. Conclusions

In conclusion, our predictions with the MMOA model, as well as available experimental data, indicate that the direct interaction of FTC-TP and TFV-DP is mainly mediated by a depletion of dNTP pools. Interactions at the level of DEC formation play a minor role for this drug combination at physiologically meaningful drug concentrations. However, experimental data suggests that DEC formation seems to be greatly facilitated when NNRTIs are added to the TDF/FTC backbone.

## Figures and Tables

**Table 1:** Microkinetic parameters. All parameters were taken from [10]. Intracellular concentrations refer to resting CD4+ T-cells (the main target of HIV) [31].

	K_D_ [μM]	k_pol_ [s^−1^]	Intracellular Concentration [μM]
dATP	7.8	44.8	1.7
dTTP	15.3	15.6	1.5
dCTP	18.25	10.2	1.9
dGTP	10.5	20	1.7
	**K_D,dNTP_** [μ**M]**	**k_pol,dNTP_** **[s^−1^]**	
TFV-DP	40.5	28	-
FTC-TP	19	0.0563	-

## Data Availability

All data necessary to reproduce the results are contained in the manuscript.

## Supplementary Materials

The following are available online at https://www.mdpi.com/article/10.3390/v13071354/s1, Table S1: Nomenclature table, Figure S1: Modulation of dNTP pools by FTC-TP and TFV-DP, Figure S2: Heatmap of Bliss independence for TFV-DP and FTC-TP interactions.
