# Supplementary material for: Mathematical Modelling of the Molecular Mechanisms of Interaction of Tenofovir with Emtricitabine against HIV
Source: Viruses. 2021 Jul 13;13(7):1354. doi: 10.3390/v13071354 (PMC8310192; doi:10.3390/v13071354)
Supplement: Supplementary file 1 [file viruses-13-01354-s001.zip › viruses-1288139-supplementary.pdf]

viruses

MDPI

Supplementary Materials: Mathematical Modelling of the Molecular Mechanisms of Interaction of Tenofovir with Emtricitabine against HIV

Table S1. Nomenclature table.

Symbol	Meaning
dNTP	Deoxynucleoside triphosphates
NRTI	Nucleotide reverse transcriptase inhibitor
$T_{0\to N}(\emptyset)$	time required to complete viral DNA polymerization in the absence of drug
$T_{0\to N}(I)$	time required to complete viral DNA polymerization in the presence of drug
N	length of viral DNA
$T_{0\to 1}$	start of recursion (from position 0 to 1 of the primer)
$T_{0\to N}$	time required to complete viral DNA polymerization
$T_{i\to i+1}$	Expected time to extend primer by one base
$\emptyset$	Absence of drug
i	Positional index along the primer
$K_{D,I}$	Dissociation constant of the NRTI
$K_{D,dNTP}$	Dissociation constant of the dNTP
k_{term}	Catalytic rate constant for the NRTI incorporation
k_{pol}	Catalytic rate constant for the dNTP incorporation
r_{term}	Rate of NRTI-TP incorporation in the primer
r_{pyro}	Pyrophosphorolysis rate
r_{pol}	Polymerase reaction
r_{exc}	Excision rate
$[dNTP]$	Endogenous dNTP concentration
$[dNTP(\emptyset)]$	basal dNTP concentration
τ	Waiting times
ρ	Jump probabilities
$\widetilde{\iota+1}$	Drug blocked state of the primer
η	inhibition of cell infection
ε	inhibition of reverse transcription by NRTI
$\rho_{\emptyset,RT}$	Probability to succeed in reverse transcription in the absence of the drug
Θ_{dNTP}	Reduction factor for the dNTP
k_{off}	Dissociation constant
K_D	Equilibrium dissociation constant
k_{on}	Association constant
IC_{50}	Concentration required for 50% inhibition
m	Hill coefficient
$Emax$	Maximum effect
$[C]$	Drug concentration
$IC_{x,1}$	Concentration of drug 1 inhibiting by x %
$IC_{x,2}$	Concentration of drug 1 inhibiting by x %
I	A drug
I_1	Drug 1
I_2	Drgu 2
$\tau_{exc}(TFV,\emptyset)$	waiting time for removal of the incorporated TFV-DP from the primer
$\tau_{exc}(TFV,FTC)$	(increased) waiting time when FTC-TP binds to the TFV-DP terminated primer
$\tau_{\widetilde{FTC}}$	probability that FTC-TP binds to the TFV-DP terminated primer

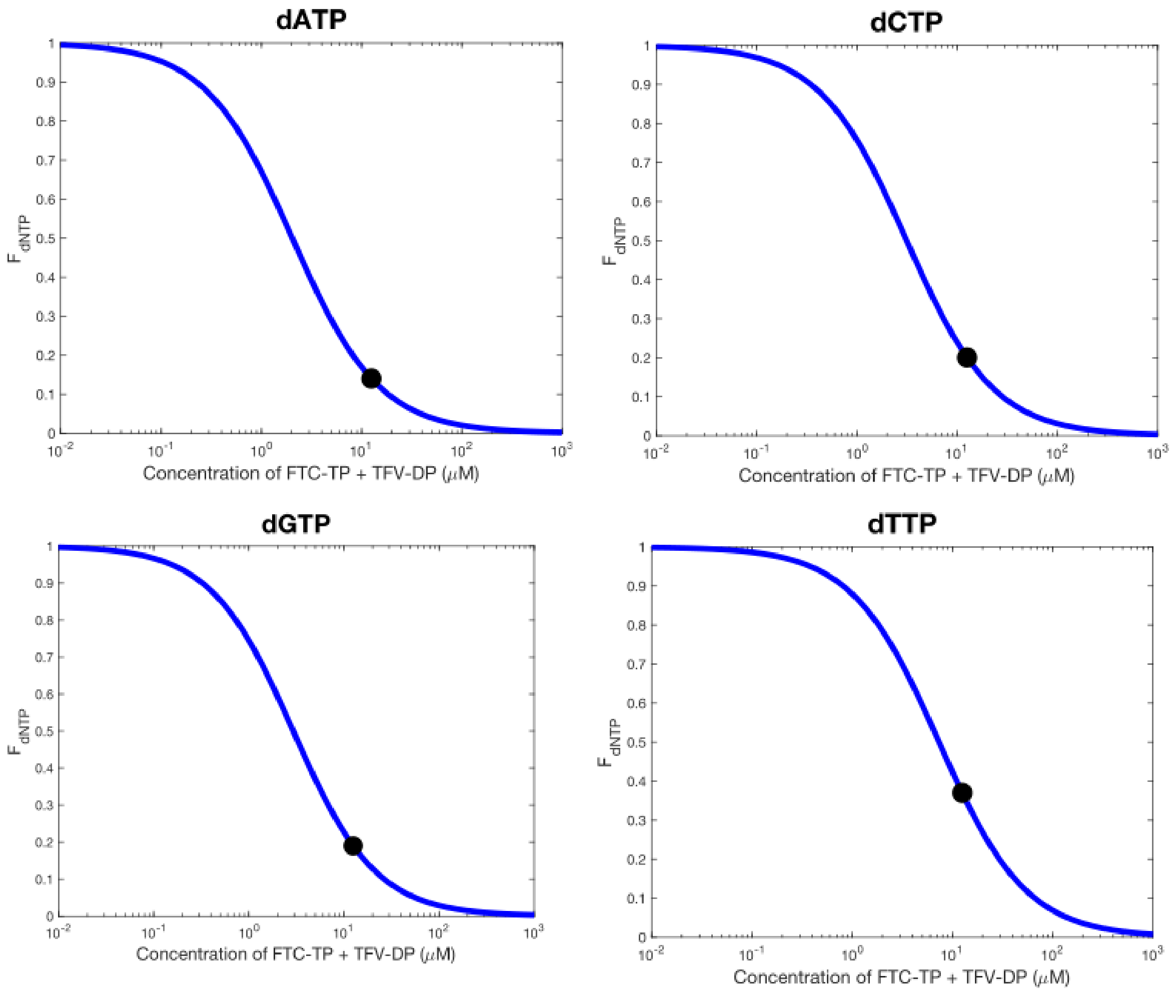

Figure S1. Modulation of dNTP pools by FTC-TP and TFV-DP. Experimentally determined [37] factors of dNTP pool reductions FdNTP after once daily treatment with 300/200mg oral TDF/FTC are depicted as black dots (in percent of basal levels: dATP: 14%, dCTP: 20%, dGTP: 19% and dTTP: 37%). In order to identify the corresponding intracellular NRTI-TP levels, we implemented the pharmacokinetic models linking the oral dosing schemes with the intracellular concentrations [29] and derived concentrations of 12.34 (FTC-TP) and 0.16μM (TFV-DP) respectively. We then fitted a continuous function for the reduction factor as depicted in eq. (11). The resulting fits are shown as solid blue lines.

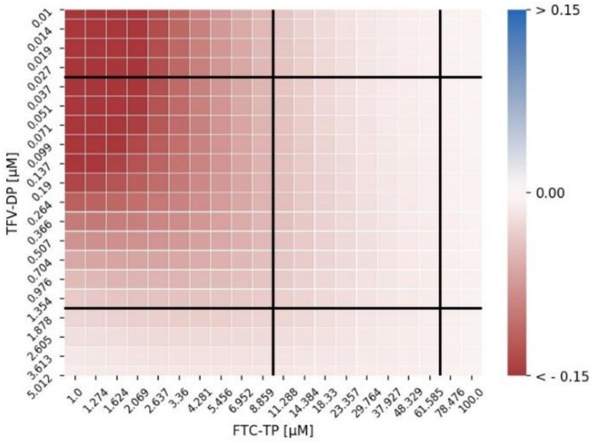

(**a**)

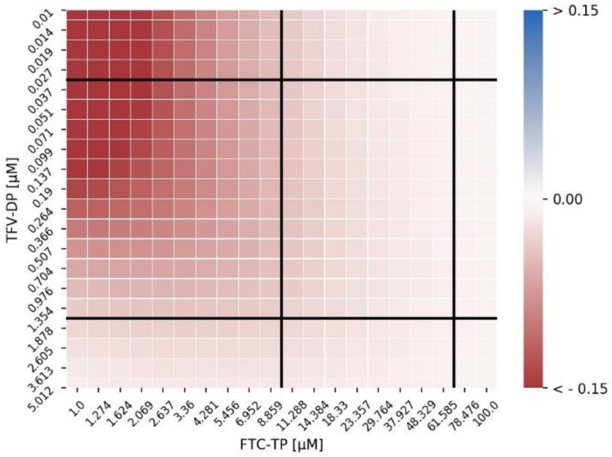

(**b**)

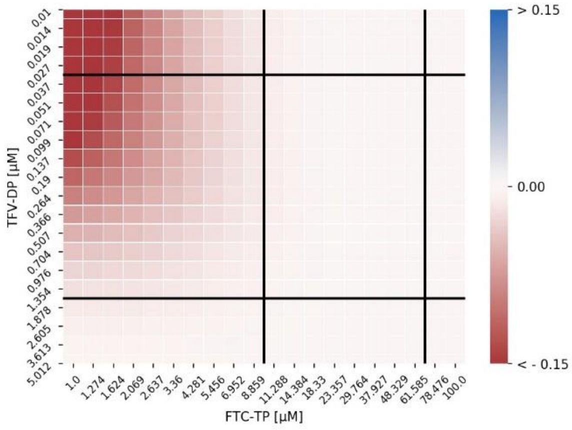

(**c**)

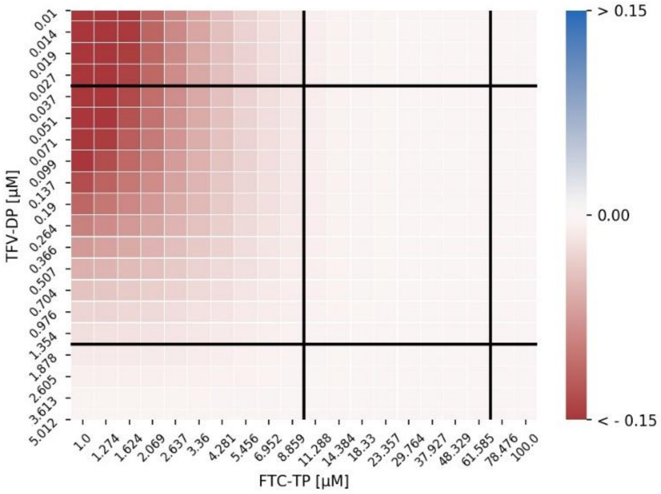

(**d**)

Figure S2. TFV-DP and FTC-TP interactions heatmaps (Bliss independence). The concentrations that produced an effect classified as synergistic are shown in blue, whereas red denotes antagonism The panels report results for the unmodified model and the other modifications applied. (**a**) control: unmodified MMOA model; (**b**) exc: decrease in the excision rate; (**c**); dNTP: reduced dNTP pools; (**d**) dNTP+ exc: reduction of dNTP and decrease in the excision rate both incorporated in the MMOA model.
